# Case Commentary: Successful Use of Cefepime/Zidebactam (WCK 5222) as a Salvage Therapy for the Treatment of Disseminated Extensively Drug-Resistant New Delhi Metallo-β-Lactamase-Producing *Pseudomonas aeruginosa* Infection in an Adult Patient with Acute T-Cell Leukemia

**DOI:** 10.1128/aac.00663-23

**Published:** 2023-07-03

**Authors:** Stephanie L. Egge, James S. Lewis, Morgan Hakki

**Affiliations:** a Division of Infectious Diseases, Department of Internal Medicine, Houston Methodist Hospital, Houston, Texas, USA; b Division of Infectious Diseases, Department of Internal Medicine, Oregon Health and Science University, Portland, Oregon, USA; c Department of Pharmacy, Oregon Health and Science University, Portland, Oregon, USA

**Keywords:** *Pseudomonas aeruginosa*, antimicrobial resistance, cefepime-zidebactam, extensively drug-resistant, metallo-β-lactamase, multidrug resistance, novel antimicrobial agents

## Abstract

Multidrug-resistant/extensively drug-resistant (MDR/XDR) Pseudomonas aeruginosa (PA) are critical antimicrobial resistance threats. Despite their increasing prevalence, treatment options for metallo-β-lactamase (MBL)-producing PA are limited, especially for New Delhi metallo-β-lactamase (NDM) producers. Pending further clinical studies, this case provides support for limited-scope use of cefepime-zidebactam for treating disseminated infections secondary to NDM-producing XDR PA. Susceptibilities should be tested and/or alternative regimens considered when treating isolates with alternative MBLs or increased efflux pump expression because some *in vitro* data suggest associated loss of cefepime-zidebactam susceptibility.

## INTRODUCTION

Pseudomonas aeruginosa (PA) is a leading nosocomial pathogen with an impressive ability to adapt under antimicrobial pressure via genomic mutations and acquisition of mobile genetic elements. Multidrug-resistant (MDR) and extensively drug-resistant (XDR) PA are serious antimicrobial resistance (AMR) threats (1, 2). XDR isolates, retaining susceptibility to only one or two antibiotic classes, are an increasing clinical concern, representing 20% to 30% of all clinical PA isolates in some geographic regions (3, 4). Both XDR and MDR PA infections are associated with mortality, which is estimated to be over 20% (5). Treatment options for MDR/XDR isolates are often limited to polymyxin-based regimens with significant toxicity and failure rates that reach 30% to 40% (4, 6, 7).

Between 2010 and 2020, both the Infectious Diseases Society of America and the Pew Trust promoted initiatives for novel antimicrobial development to meet the challenge of rapidly growing AMR threats such as MDR/XDR PA. The Generating Antibiotics Incentives Now (GAIN) Act and the “10 by ’20 Initiative” supported the development of 14 potential anti-pseudomonal therapies (8, 9). Four of these drugs have achieved FDA approval and represent treatment options for MDR/XDR PA: the novel cephalosporins ceftolozane (combined with tazobactam) and cefiderocol and two diazabicyclooctane beta-lactamase inhibitor-based combinations, ceftazidime-avibactam and imipenem-relebactam (10). Despite the activity of all four novel anti-pseudomonal agents against many carbapenem-resistant PA isolates, ceftolozane-tazobactam, imipenem-relebactam, and ceftazidime-avibactam are rendered inactive by metallo-β-lactamase (MBLs) (11).

In this report, Tirlangi et al. outline a case of bloodstream infection with associated pulmonary and necrotizing soft tissue involvement in an immunocompromised patient due to an XDR P. aeruginosa infection resistant to all carbapenems, ceftazidime-avibactam, and ceftolozane-tazobactam. The authors did not have access to cefiderocol as a treatment option. The patient exhibited signs of worsening sepsis and neurotoxicity while receiving polymyxin B plus meropenem therapy, necessitating the exploration of alternative therapies.

Although Kirlangi et al. did not have access to cefiderocol or associated-testing, they list several reasons to consider alternatives to this last-line approved anti-pseudomonal agent. First, they note the detection of a New-Delhi metallo-β-lactamase (NDM). While cefiderocol has promising verona integron-encoded metallo-β-lactamase (VIM) and imipenemase-type metallo-β-lactamase (IMP) *in vitro* activity, various studies have cited exogeneous MBL and serine β-lactamase-associated cefiderocol resistance across *Enterobacterales* and non-lactose fermenter Gram-negatives, including Pseudomonas aeruginosa and Acinetobacter baumannii (12–14). Furthermore, even in the absence of MBLs, increasing reports of resistance to ceftolozane-tazobactam, ceftazidime-avibactam, and cefiderocol have been seen in Europe, the Americas, and Asia (12, 15–17). It has been demonstrated that isolates with ceftazidime-avibactam and ceftolozane-tazobactam resistance via AmpC/pseudomonas-derived cepharolsporinase (PDC) point mutations may present a high risk for the development of additional AmpC/PDC mutations that confer cefiderocol non-susceptibility (17). Diminished cefiderocol susceptibility and/or heteroresistance has also been associated with *de novo* mutations in PBP3 and TonB-dependent receptors PiuA/PirA (16, 18). Importantly, susceptibility testing of cefiderocol remains challenging, making it difficult to determine which isolates will respond to cefiderocol therapy (19, 20).

Cefepime-zidebactam (WCK 5222) is a novel bicyclo-acyl hydrazide-containing combination agent that is under investigation for complicated urinary tract infection or pyelonephritis (21). In addition to inhibiting class A, class C, and some class B β-lactamases, zidebactam acts as an “enhancer” with intrinsic antimicrobial activity via high-affinity binding to PBP2 (22). Limited *in vitro* data suggest that cefepime-zidebactam maintains activity against MDR and XDR P. aeruginosa, including MBL-producing isolates (11, 22–25).

It should be noted that higher cefepime-zidebactam MICs have been reported in some isolates with efflux pump overexpression (i.e., mexAB-oprM or mexXY) and PBP3 amino acid substitutions, and in the setting of some VIM or IMP-type MBLs (18, 26). Given these limited data, cefepime-zidebactam should be reserved for the treatment of MDR/XDR PA isolates harboring porin loss and certain class A, C, and select metallo-β-lactamases, and antimicrobial susceptibility testing should guide its use. Other novel agents might be a more ideal choice for the treatment of non-MBL-mediated resistance involving efflux pump overexpression. Lastly, the susceptibility criteria proposed by the authors have not been evaluated by breakpoint setting organizations and are higher than those currently in place for a 2-g-every-8-h regimen of cefepime (27).

In this clinical case with the associated resistance markers, cefepime-zidebactam was a logical and promising treatment option, especially since the authors did not have access to cefiderocol. Not only was cefepime-zidebactam successful at eradicating an infection in an immunocompromised patient that failed to respond to prior antibiotic therapies, but it was also well tolerated. The authors should be commended for their efforts to obtain cefepime-zidebactam on a compassionate-use basis, which almost certainly saved their patient’s life. Their lack of access to cefiderocol also highlights the need to make novel agents more available in areas where the burden of MDR and XDR infections is greatest and they are most needed.

Cases such as the one described here are becoming more common, specifically in Asia. However, these worrisome organisms also appear increasingly common in the United States and Europe (28). Although the current global prevalence of these infections remains low, this limits our ability to rapidly assess the microbiologic and clinical efficacy of newer agents for these highly challenging infections. In the interim, while we await more definitive controlled-data and randomized-control trials, this case provides valuable insight into a seemingly safe and effective treatment option for XDR PA (29).
